# Effects of transcutaneous electric nerve stimulation on pain after episiotomy. A systematic review and meta-analysis

**DOI:** 10.1016/j.heliyon.2024.e41577

**Published:** 2024-12-31

**Authors:** Elisa López-Campos, Mercedes Soto-González, Alejandra Alonso-Calvete, Iria Da Cuña-Carrera

**Affiliations:** a Faculty of Physiotherapy, University of Vigo, Pontevedra, Spain; b Clinical Physiotherapy Group, Galicia Sur Health Research Institute (IIS Galicia Sur) SERGAS, Vigo, Spain; c REMOSS Research Group, University of Vigo, Pontevedra, Spain

**Keywords:** Physiotherapy, Episiotomy, Transcutaneous electric nerve stimulation, Pain

## Abstract

**Background:**

Episiotomy is a surgical intervention performed during the second stage of labor to facilitate the baby's exit through the birth canal. There are different reasons that lead **to an** episiotomy; however, it is recommended **to be** performed occasionally and not systematically, since it may produce negative effects such as pain. Different therapies have been described to reduce this pain, including transcutaneous electrical nerve stimulation (TENS).

**Objective:**

To conduct a systematic review and meta-analysis about the effects of Transcutaneous Electric Nerve Stimulation (TENS) on pain after episiotomy.

**Methods:**

The databases PubMed, Cochrane, Scopus, Cinhal, PEDro and Web of Science were consulted using the terms “Episiotomy” and “Transcutaneous Electric Nerve Stimulation”. The methodological quality was analyzed with the PEDro scale. A random-effects model was used to carry out the meta-analysis.

**Findings:**

88 studies were obtained and after applying the selection criteria, 6 were included in the systematic review and 5 in the meta-analysis. There were significant positive effects in decreasing the pain for TENS in comparison with pre-intervention (SMD = 1.19 [95 % CI – 0.33 to 2.05]; p < 0.01; I2 = 96 %), control group (SMD = −1.07 [95 % CI – −1.53 to −0.6]; p < 0.01; I2 = 82 %) and placebo group (SMD = −1.33 [95 % CI – −2.32 to −0.34]; p < 0.01; I2 = 86 %).

**Conclusion:**

TENS appears to have a positive effect in reducing pain after 1 h of an episiotomy. The location of the electrodes does not seem to be relevant in the effects.

## 1 Introduction

Episiotomy is a surgical intervention performed on the pelvic floor musculature during the second stage of labor, with the aim of widening the birth canal and thus facilitating the exit of the baby's head [1]. This incision may have different shapes. The most commonly used is the mediolateral incision because it is the least damaging and easiest to perform, with an angle of 45°-50° [[2], [3], [4]].

Different reasons may lead to the performance of this intervention. Many of them are influenced by the context of delivery, which are associated both with the characteristics of the mother and the fetus, as well as the delivery itself. The mother's age, number of previous deliveries and episiotomies, ethnicity and fetal status, length of the second stage and the use of instruments during this phase are among the most studied factors. It is more frequently used in teenage mothers and females over 35 years of age, primiparous women, Asians, if fetuses are over 3,500g or in acute fetal distress, in long second stages where the mother is not able to push efficiently, and in deliveries where the use of instruments such as the vacuum cup is necessary. In fact, it may prove to be a protective technique for some complications derived from instrumentalized deliveries, such as visceral prolapse [2,5,6].

Both the WHO and various National and International Health Organizations recommend the use of episiotomy on an exclusively punctual basis only, on those cases where it can be beneficial, such as those described above. However, they do not support its systematic use, since its effects are more negative than positive, such as higher grade tears and pelvic floor dysfunctions (pain, dyspareunia, muscular atrophy, incontinence, etc.) [[7], [8], [9]]. There is a tendency to decrease its routine use worldwide. However, this practice is still very present today [10,11].

The negative effects of episiotomies affect the patients’ quality of life and health, both in the short and the long term. Pain is one of the main problems, which is sometimes associated with dyspareunia. This occurs in the postpartum period and lead to problems on normal sexual activity [12]. To a lesser extent, incontinence occurs due to damage to the vaginal wall and anal sphincter, followed by dehiscence of the stitches and wound infections, episiotomy may be related with an increased risk for multiparous, for obstetrical anal sphincter injury [13] Moreover, trauma to the pudendal nerves during episiotomy may increase the effect of direct sphincter injuries [14].

There are different techniques in addressing the pain caused by episiotomy. The most commonly used are cryotherapy in the hours following episiotomy, manual therapy and scar management so as to avoid tissue adhesions, as well as exercise [[15], [16], [17], [18]].

In addition, some studies suggest electrotherapy, specifically transcutaneous electrical nerve stimulation (TENS) because of the effects it has on the tissues [16,[19], [20], [21]]. However, to date and to the best of authors’ knowledge, no prior systematic reviews and meta-analyses have analyzed the effects of TENS on pain after episiotomy. Therefore, the aim of this study is to review the scientific literature about the effects of TENS on episiotomy pain.

## 2 Methods

A systematic review and meta-analysis was performed with the Preferred Reporting Items for Systematic Reviews and Meta-Analyses (PRISMA) guidelines [22] in September 2024. The code for the PROSPERO registration is CRD42023479075.

### 2.1 Search strategy

A systematic search was carried out according to the PICO question in the databases: *PubMed, Cochrane, Scopus, Cinhal, Web of Science and PEDro.* A different combination of terms in relation to “Episiotomy”, “Transcutaneous Electric Nerve Stimulation” and “Pain” was used in the search strategy. The search strategy adapted for each database and the results of each search can be found in the Supplementary Data.

### 2.2 Eligibility criteria

The purpose of the study and the eligibility criteria were performed according to the PICO question (Participants, Interventions, Comparators and Outcomes) [23]. The participants were women with episiotomy during labor, the intervention was TENS, no comparisons were analyzed, and the main outcome was pain.

The inclusion criteria were randomized clinical trials (RCT) which analyzed the effects of TENS on the pain of episiotomy. The exclusion criteria were studies in other types of perineal injuries or different electrotherapy methods.

### 2.3 Study selection and data extraction

Two of the investigators (A.A.C and M.S.G) conducted the screening, eligibility, and extraction of the data independently, to avoid potential bias. In case of disagreement, a third researcher (I.D.C) was consulted. This process was based on the minimum requirements of Cochrane for Inclusion and Exclusion Criteria [24].

### 2.4 Methodological quality

The PEDro scale was selected to analyze the methodological quality of the studies. This scale assess the quality of intervention studies with 11 items which evaluate potential sources of bias, giving one point to the study if it clearly satisfies the criteria. The score reached in each study is calculated by summing the score of 10 of the items, classifying the studies as excellent (10-9), good (8–6), medium (5–4) or poor (3–0). The first item does not count to the final score [25].

Risk of bias was analyzed with “The Cochrane Collaboration Tool”, which examine different bias and classify them into “high risk”, “low risk” or “unclear”(26).

### 2.5 Statistical analysis

The statistical analysis was carried out using Comprehensive Meta-Analysis software version 2.2.064 for Windows (Biostat Inc., Englewood, New Jersey, United States). Random effects models were conducted to determine and compare the effects of TENS on pain pre and post intervention, in comparison with a placebo intervention and in comparison, with a control group. To estimate the magnitude of TENS intervention, standard mean differences (SMD) values with 95 % confidence intervals were used. The SMD were read as trivial (SMD<0.2), small (0.2≥SMD<0.5), moderate (0.5 ≥ SMD <0.8), or large (SMD ≥0.8) [27]. The significance level was set at p < 0.05 and heterogeneity was evaluated using the I^2^ statistic, to show the percentage of variation in estimated effects across studies, due to heterogeneity rather than chance. This I^2^ was interpreted as low (I^2^ < 25 %), moderate (25 % ≥ I^2^ < 75 %), and high (I^2^ ≥ 75 %) [24].

## 3 Results

### 3.1 Study selection

The systematic search conducted in this study reported 88 articles. After applying the eligibility criteria, a total of 6 studies were finally included in this systematic review. Initially, all studies met the inclusion and exclusion criteria for the quantitative synthesis with meta-analysis but after an in-depth analysis of the designs of the studies and the report of the results, the investigation of Lorenzana et al. [28] has been excluded since they do not provide pre and post-test measurements so meta-analysis could not be conducted.

**Fig. 1:**
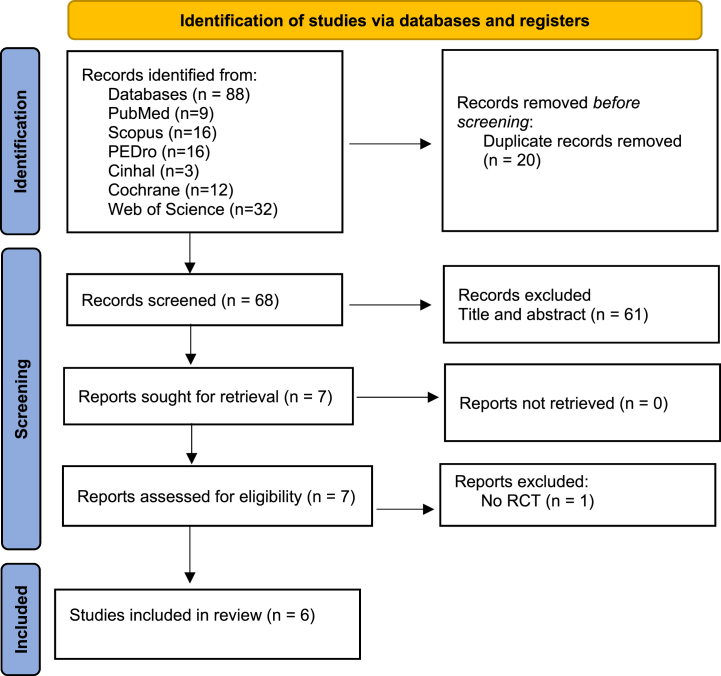
Flow chart according to PRISMA.

The process of the systematic search is described in Fig. 1, according to the flow chart described in PRISMA guidelines [22].

### 3.2 Characteristics of the studies

The main characteristics of the studies in this systematic review and meta-analysis are detailed in Table 1.

**Table 1:** Characteristics of the studies.

Autor	Sample	Age (years)	Episiotomy	Groups	Measurements	Results
Zakariaee et al. [29]	120	+18	Mediolateral	G1: placebo G2: control (usual treatment) G3: TENS	*Pain:* register form - VAS.	G3 ↓ pain just after and 1h after in the resting and walking positions. There was no difference in sitting.
					*TENS satisfaction form*.	
Pitangui et al. [30]	40	18–31	–	G1: control (usual treatment) G2: TENS	*Pain*: NPRS scale and McGill Pain Questionnaire	G2 ↓ pain after 60 and 120 min
					*TENS satisfaction form.*	50 % high satisfaction, 50 % medium satisfaction
Pitangui et al. [32]	33	+15	Media o mediolateral	G1: TENS HF G2: TENS LF G3: placebo	*Pain*: NPRS scale	G1 = G2. G1 y G2 ↓ pain vs G3. Significant differences at 60 and 120 min.
					*TENS satisfaction form*.	
Lorenzana et al. [28]	72	26–27	Media	G1: control + extra lidocaine G2: TENS	*Pain*: VAS	G2 ↓ pain after 1h 37 % of women needed rescue anesthesia in G2
					*Tissue condition (vaginal exam)*	G2 ↓ edema, inflammation, and sensibility.
Rezaeyan et al. [40]	80	18–35	Mediolateral	G1: control + extra lidocaine G2: TENS	*Pain*: VAS	G2 ↓ pain after 1h and 12h
					*Tissue condition (REEDA scale)*	G2 ↓ edema
Mehri et al. [42]	130	24	–	G1: control + extra lidocaine G2: TENS	*Pain*: VAS	G2 ↓ pain just after and 1h after

NPRS: Numerical Pain Rating Scale, G: group. Questionnaire. TENS: Transcutaneous Electric Nerve Stimulation REEDA: Redness, Edema, Ecchymosis, Discharge, Approximation, VAS: Visual Analog Scale, ↓: significant decrease in pain, HF: high frequency, LF: low frequency.

All the investigations included analyzed women with episiotomy during labor, with a range age between 15 and 35 years old. The main type of episiotomy was the mediolateral [[29], [30], [31]] or the medium episiotomy [28,30],and only two studies did not report this information [32,33]. The investigations were conducted in Irán [29,31,33], Brazil [30,32] and Philippines [28]. All the studies analyzed pain as the main outcome of the intervention [[28], [29], [30], [31], [32], [33]], but other variables were included such as the tissue condition (edema) [28,31], the satisfaction with the therapy [29,30,32]and function [32].

Moreover, the specific values of the TENS application are reported in Table 2. It is remarkable that all studies used the same frequency (100 Hz) for the application of TENS after the episiotomy. Besides, the applications were cutaneous and at different moments, with all the studies analyzing 60 min post episiotomy.

**Table 2:** TENS parameters.

Author	Electrodes	Area of application	Frequency (Hz)	Pulse (uSec)	Time (min)
Zakariaee et al. [29]	4 silicone electrodes	Parallel to the episiotomy	100	75	30, 60, 120
Pitangui et al. [41]	4 silicone electrodes	Parallel to the episiotomy	100	75	Pre, 60, 120
Pitangui et al. [39]	2 silicone electrodes	Parallel to the episiotomy	100	100	Pre, 30, 60, 120
Lorenzana et al. [37]	2 cutaneous electrodes	2 acupuncture points: LI-4 “Hegu” y H-7 “Senmen”	100	2–100	Pre, during, 60, 360
Rezaeyan et al. [40]	2 cutaneous electrodes	2 acupuncture points: LI-4 “Hegu” y H-7 “Senmen”	100	250	Pre, during, 60
Mehri et al. [42]	4 cutaneous electrodes	4 acupuncture points: LI-4 “Hegu”, H-7 “Senmen”, SP6 “Sanyinjiao” and extra “Neimadian”	100	–	60, 720

Hz: Hertz. LI-4 Hegu, H-7 Senmen, SP6 Sanyinjiao, extra Neimadian: points described in acupuncture techniques. Min: minutes. uSec: pulse per second.

### 3.3 Methodological quality

The scores reached in the PEDro scale are described in Table 3. All studies reported an excellent quality except from Mehri et al. [33] with a good quality. The Item 6 was not satisfied in any study and item 7 in only one study [28].

**Table 3:** Results of the PEDro scale.

Author	Item 1	Item 2	Item 3	Item 4	Item 5	Item 6	Item 7	Item 8	Item 9	Item 10	Item 11	Total
Zakariaee et al. [38]	1	1	1	1	1	0	0	1	1	1	1	**9**
Pitangui et al. [41]	1	1	1	1	1	0	0	1	1	1	1	**9**
Pitangui et al. [39]	1	1	1	1	1	0	0	1	1	1	1	**9**
Lorenzana et al. [37]	1	1	1	1	1	0	1	1	1	1	1	**10**
Rezaeyan et al. [40]	1	1	1	1	1	0	0	1	1	1	1	**9**
Mehri et al. [42]	1	1	0	1	1	0	0	1	1	1	1	**8**

Item 1: eligibility criteria; Item 2: random allocation; Item 3: Concealed allocation; Item 4: Baseline comparability; Item 5: Blind subjects; Item 6: Blind therapists; Item 7: Blind assessors; Item 8: Adequate follow-up; Item 9: Intention-to-treat analysis; Item 10: Between-group comparisons; Item 11: Point estimates and variability.

**Fig. 2:**
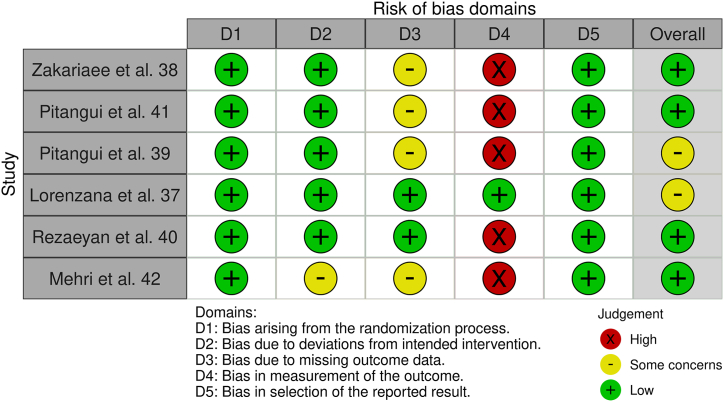
Analysis of risk of bias (Robvis).

About the results of the analysis of risk of bias with “The Cochrane Collaboration Tool” [26], results are detailed in Fig. 2. It is remarkable the high risk of detection bias (item 4), but there are low risk in the randomization processes (item 5).

### 3.4 Effects of TENS on pain pre and post intervention

Five studies compared the level of pain before and after an intervention with TENS(29–33). All studies measured pain with a VAS scale and performed the measurements 1 h after the episiotomy in resting position. The interventions lasted from 30(30) to 60 min [29,[31], [32], [33]] and at the end, pain was measured again.

**Fig. 3:**
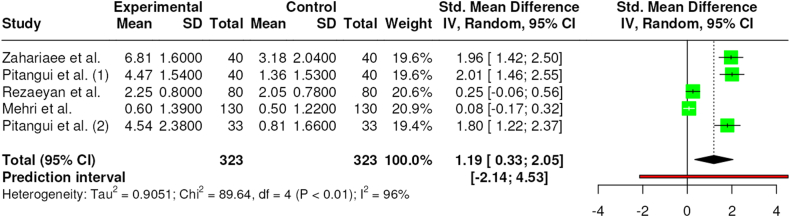
Effects of TENS on pain pre and post intervention.

As a result of this intervention, all studies reported a significant decrease of pain after the TENS intervention in comparison with the pre-test measurements. Moreover, findings of the meta-analysis reported significant effects between pre and post intervention (SMD = 1.19 [95 % CI – 0.33 to 2.05]; p < 0.01; I^2^ = 96 %) on pain. The relative weight of each study in the analysis varied between 20.9 % and 19.4 %, indicated by the size of the plotted box in Fig. 3.

### 3.5 Effects on TENS in comparison with control group

Four studies analyzed the level of pain after the TENS intervention vs a control intervention based on lidocaine on the area of the episiotomy [29,31,33]. Again, all the interventions were conducted 1 h after the episiotomy, in resting position and pain was measured in all of them with a VAS scale. The duration of the interventions varied from 30(30) to 60 min [29,31,33].

**Fig. 4:**
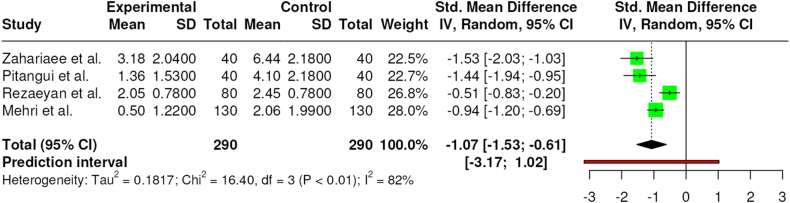
Effects on TENS in comparison with control group.

All studies showed a significant decrease of pain with the TENS intervention in comparison with the control intervention. The results of the meta-analysis demonstrated significant differences between the two interventions (SMD = −1.07 [95 % CI – −1.53 to −0.6]; p < 0.01; I^2^ = 82 %) with a positive effect for the TENS intervention in the decrease of pain. The relative weight of each study in the analysis changed between 28 % and 22.5 %, indicated by the size of the plotted box in Fig. 4.

### 3.6 Effects of TENS in comparison with a placebo intervention

Two investigations compared the TENS intervention with a placebo intervention based on the same application but with the device turned off [29,32]. All interventions were conducted 1 h after the episiotomy in resting position. Pitangui et al. [32] performed a 30-min intervention and Zahariaee et al. [29] a 60-min intervention. Pain was measured in both intervention with a VAS scale.

**Fig. 5:**
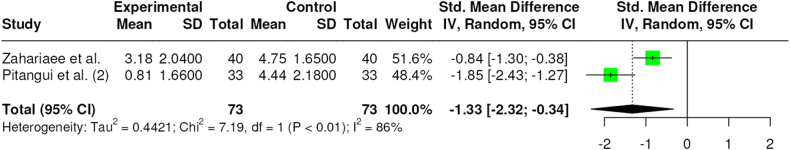
Effects of TENS in comparison with a placebo intervention.

Both investigations reported a decrease of pain with the TENS intervention in comparison with placebo. The results of the meta-analysis showed a significant decrease of pain after the TENS intervention in comparison with placebo (SMD = −1.33 [95 % CI – −2.32 to −0.34]; p < 0.01; I^2^ = 86 %). The relative weight of each study in the analysis varied between 48.4 % and 51.6 %, showed in the size of the plotted box in Fig. 5.

## 4 Discussion

The aim of this systematic review and meta-analysis was to analyze the effects of electrotherapy on episiotomy pain.

Pain is a central feature of parturition in women, and its relief is essential for medical care during labor [34]. TENS is used throughout the world for symptomatic relief of pain, supported by physiological evidence that TENS inhibits the activity and excitability of central nociceptive transmission neurons, irrespective of diagnosis [35]. The physiological effects of this technique are based on the Gate Control Theory, to justify the analgesic effect of this type of current. This theory is based on the interference with pain transmission by another non-painful stimulus due to the existence of interneurons [36,37]. Previous research [31] mentions two additional theories, which are the Diffuse Noxious Inhibitory Control Theory [38] and the Mediation of Pain. Another article [33], also justifies the modulation of pain by means of the Qi theory related to acupuncture [[39], [40], [41]].

The results of our meta-analysis reported significant effects between before and after the intervention on pain, thus confirming that TENS has an analgesic effect on episiotomy pain.

As for the meta-analysis on the comparison of pain between the intervention and control groups, all the studies showed a significant decrease in pain for those patients who added TENS to the usual treatment based on local anesthesia with lidocaine, cold packs and analgesic medication. Therefore, it would be beneficial to consider adding this therapy at this stage since several studies showed that combining TENS oral analgesia and localized cooling achieved the greatest relief of perineal pain, in comparison with the use of only localized cooling [42].

Regarding the use of anesthetics in the studies, two compare the application of TENS with an additional dose of lidocaine. As a result, it is shown that the analgesic effects of TENS may be an additional advantage when sewing the episiotomy, due to its subsequent anti-inflammatory effect [28,31].

It should be noted that the exclusion of patients receiving epidural analgesia or similar was determined in two studies [29,32] Another study [32] only records or mentions the analgesics consumed and their possible amounts. Finally, another study [33] does not record the analgesics, but it is known that they are administered to the patients. In the case of the last two [32,33]there is no differentiation or mention to the possibility of their interference on the perceived pain that the presence of the analgesics in the patient's body may entail. Hence, this could translate into a possible confounding bias in the results, which could lead to a lower internal validity of the studies.

Despite this, all the articles have a control or placebo group that receives analgesics and a treatment group, so that any difference in perceived pain could be considered as the effect of the therapy application.

Two investigations have compared the TENS intervention and the TENS placebo [29,30] and both reported a decrease in pain with the TENS intervention compared to placebo, which demonstrates again the positive effect of this therapy on episiotomy pain. These results coincide with the results obtained in the meta-analysis by Johnson et al. [35] who found that pain intensity was lower during or immediately after the administration of TENS on painful body parts compared to placebo.

When analyzing the different studies, there are certain variables that should be taken into account. Samples of the articles have been compared, and they include women whose episiotomies and deliveries have different characteristics, which could influence their pain and recovery. Regarding the type of episiotomy (mediolateral [[29], [30], [31]], medial [28,30] or not specified [32,33], there is no uniformity in choosing the type of episiotomy despite the fact that mediolateral and lateral incisions are known to be the least harmful to the pelvic floor structure, and their possible influence on perceived pain is not considered as an aspect to be taken into accountt [3], in several studies [32,33]. Regarding the size of the incision, only one article [28] collects information on the length of the episiotomy, the average being between 3 and 4 cm. Other support the possibility that the length of the episiotomy is not of marked relevance for pain, but that its influence is centered on other associated complications such as incontinence or infections. Thus, and in contrast with previous research, Kalis et al. [43] indicate that mediolateral episiotomy produces a larger scar and requires more analgesia than others, so these variables should be taken into account.

As for the number of episiotomies or previous deliveries, they have been mentioned in four articles [28,29,32,33] where no distinction is made except for one of them [33] that includes only primiparous women. Multiple physiological and psychological factors are related to pain. Anxiety may play an important role in the experience of labor pain [44] As Green et al. [45], state, Primiparous women reported higher levels of fear, lack of control, and dissociation emotions compared to multiparous women. This aspect should be considered, since it can influence the perception of pain and, subsequently, the treatment results. Out of all the articles, only one of them [33] makes a centralized application to primiparous women and none of them to multiparous women only, so it is not possible to compare this aspect or to deduce that there is a connection with the results on pain.

When comparing the therapy application area, it is divided into direct and indirect application. For three of the studies [29,30,32], the application is direct over the episiotomy or parallel to it. For the remaining studies [28,31,33] the application is indirect over acupuncture points ‘Hegu’, ‘Shenmen’, ‘Sanyinjiao’ and ‘Neimadian’, whose effect is generally on pain and anxiety [40,41,46,47], but does not have a specific effect on the perineum or uterus as other points do, such as ‘Zhi Yin”[48]. Therefore, regarding TENS studies, it is applied both directly and indirectly, thus obtaining a greater decrease in pain in the treatment group than in the control group in both cases. In the study by Levin [49], the effect of conventional TENS versus acupuncture TENS on afferent neuronal fibers is studied, obtaining a decrease in pain with both techniques. In another study by Mehendale et al. [50] which also compares these two modalities in C-sections, concludes positive and similar effects of both types of stimulation on pain. Thus, the area of application does not seem to significantly influence the results related to pain. However, we should point out that, although remote application shows good results, studies refer to the need for rescue anesthesia in a third of the sample. Therefore, it would be necessary to compare both placings in order to rule out or confirm the inferiority of this placing with respect to direct application.

As for the application times, we observe that it can be at the time of the episiotomy [28,31,33], between 6 and 24 h after the episiotomy [29,30,32], the time with the most evidence being some hours after the episiotomy (although there are studies that state that 6 h is the recommended time to begin activities for postpartum women, and 24 h is the peak time of pain after episiotomy [51]). There are studies that use electrotherapy in women with both tears and episiotomies, which focus on long-term treatment, such as that of Peng et al. [52] with TENS, whose application is 6–8 weeks after delivery, or that of Sumian et al. [53] with electrical stimulation, which performs treatments from 2 days to 3 months post-episiotomy. However, a greater number of articles are found with short-term electrotherapy applications, such as that of Foulkes [54], therefore it could be deduced that the evidence that currently exists focuses on the use of electrotherapy as an analgesic method in acute episiotomy pain, i.e., it would focus on the immediate effect of TENS on episiotomy.

Regarding the TENS parameters, the different characteristics of each therapy are analyzed with the aim of studying their possible influence on the results obtained on pain.

Regarding the methodological quality of the articles, which was analyzed using the PEDro [25] scale, we found that all of them are of excellent quality [[28], [29], [30], [31], [32]] except for one [33], which is of good quality. Something similar occurs with the risk of bias analyzed with The Cochrane Collaboration [26] in the articles, since the only bias with a high risk is that of detection, which is shared among all the therapies. Therefore, in case it had an influence on the therapy applications, it would have an influence on all of them and not on one over the other. Therefore, it would not be decisive when justifying the difference in the results on pain.

Results of this systematic review and meta-analysis suggest that the effects do not vary between the immediate application and 120 min. However, reductions in pain can only be demonstrated up to 120 min.

As limitations, there was a scarcity of scientific evidence on the application of electrotherapy for episiotomy pain, this being one of the first reviews carried out on the subject. Due to this scarcity of scientific articles on the subject, it was decided not to limit the review to more current studies, and articles dating from 1999 to 2022(28–33). were finally included. Even so, we wanted to preserve the level of scientific evidence to randomized clinical studies, which have high scores in different systems of analysis of the level of evidence.

As future lines of study, we would recommend conducting studies that consider the type and status of the episiotomy, number of deliveries and previous episiotomies, and the avoidance/control of the presence of analgesic medications at the time of therapy application. Moreover, future studies should consider performing longer applications of TENS in episiotomy to analyze the long-term effects.

## 5 Conclusion

TENS appears to immediately reduce episiotomy pain. In addition, the results on the tissue condition are inconclusive.

The placement of the TENS electrodes is not a determining factor in reducing pain, with positive results being obtained in its local application or by means of acupuncture points.

## CRediT authorship contribution statement

**Elisa López-Campos:** Supervision, Project administration, Formal analysis. **Mercedes Soto-González:** Writing – review & editing, Writing – original draft, Data curation. **Alejandra Alonso-Calvete:** Validation, Investigation, Funding acquisition. **Iria Da Cuña-Carrera:** Resources, Investigation, Conceptualization.

## Data and code availability

The authors are unable or have chosen not to specify which data has been used.

## Funding

This research did not receive any funding

## Declaration of competing interest

The authors declare that they have no known competing financial interests or personal relationships that could have appeared to influence the work reported in this paper.
