# Supplementary material for: Effects of transcutaneous electric nerve stimulation on pain after episiotomy. A systematic review and meta-analysis
Source: Heliyon. 2024 Dec 31;11(1):e41577. doi: 10.1016/j.heliyon.2024.e41577 (PMC11754501; doi:10.1016/j.heliyon.2024.e41577)

1. SEARCH EQUATIONS

| PubMed | ("Episiotomy"[Mesh] OR "Episiotomy") AND ("Transcutaneous Electric Nerve Stimulation"[Mesh] OR "Transcutaneous Electric Nerve Stimulation" OR "Transcutaneous Nerve Stimulation" OR "TENS" OR "transdermal electrostimulation" OR "analgesic cutaneous electrostimulation" OR "electroanalgesia" OR "neuromodulation" OR "Neuromuscular electrical stimulation" OR "NMES" OR "electrical muscle stimulation" OR "EMS" ) AND ("Pain"[Mesh] OR "Pain" OR "Acute Pain"[Mesh] OR "Acute pain" OR "Pain Management"[Mesh] OR "Pain management" OR "Nociceptive Pain"[Mesh] OR "Nociceptive pain" OR "Childbirth pain") |
| --- | --- |
| Cochrane | **#1: Episiotomy**  #2: Transcutaneous Electric Nerve Stimulation  #3: Transcutaneous Nerve Stimulation  #4: TENS  #5: transdermal electrostimulation  #6: analgesic cutaneous electrostimulation  #7: electroanalgesia  #8: neuromodulation  #9: Neuromuscular electrical stimulation  #10: NMES  #11: electrical muscle stimulation  #12: EMS  **#13: #2 OR #3 OR #4 OR #5 OR #6 OR #7 OR #8 OR #9 OR #10 OR #11 OR #12**  #14: Pain  #15: Acute Pain  #16: Nocioceptive pain  #17: Pain Management  #18: Childbirth  **#19: #14 OR #15 OR # 16 OR #17 OR #18**  **#20: #1 AND #13 AND #19** |
| Scopus | ( TITLE-ABS-KEY ( episiotomy ) AND TITLE-ABS-KEY ( "Transcutaneous Electric Nerve Stimulation" OR "Transcutaneous Nerve Stimulation" OR "TENS" OR "transdermal electrostimulation" OR "analgesic cutaneous electrostimulation" OR "electroanalgesia" OR "neuromodulation" OR "Neuromuscular electrical stimulation" OR "NMES" OR "electrical muscle stimulation" OR "EMS" ) AND TITLE-ABS-KEY ( "Pain" OR "Acute pain" OR "Pain management" OR "Nociceptive pain" OR "Childbirth pain" ) |
| Cinhal | (MH "Episiotomy" OR “Episiotomy”) AND (MH “transcutaneous electric nerve stimulation” OR "Transcutaneous Electric Nerve Stimulation" OR “Transcutaneous Nerve Stimulation” OR "TENS" OR “transdermal electrostimulation” OR “analgesic cutaneous electrostimulation” OR “electroanalgesia” OR “neuromodulation” OR “Neuromuscular electrical stimulation” OR “NMES” OR “electrical muscle stimulation” OR “EMS”) AND (MH "Pain" OR ”Pain” OR “Acute pain” OR MH "Nociceptive Pain" OR “Nociceptive Pain” OR MH "Pain Management" OR “Childbirth pain”) |
| PEDro | "episiotomy"- Selected in "therapy": "electrotherapies, heat, cold"- Selected in “problem” “pain”. |
| Web of Science | #1: TS=(episiotomy)  #2: (((((((((((TS=(Transcutaneous Electric Nerve Stimulation)) OR TS=(Transcutaneous Nerve Stimulation)) OR TS=(TENS)) OR TS=(transdermal electrostimulation)) OR TS=(analgesic cutaneous electrostimulation)) OR TS=(electroanalgesia))) OR TS=(neuromodulation)) OR TS=(Neuromuscular electrical stimulation)) OR TS=(NMES)) OR TS=(electrical muscle stimulation)) OR TS=(EMS)  #3: ((((TS=(Pain)) OR TS=(Acute pain)) OR TS=(pain management)) OR TS=(nocioceptive pain)) OR TS=(childbirth pain)  **#4: #3 AND #2 AND #1** |

1. **SEARCH RESULTS**

PUBMED (9 RESULTADOS)


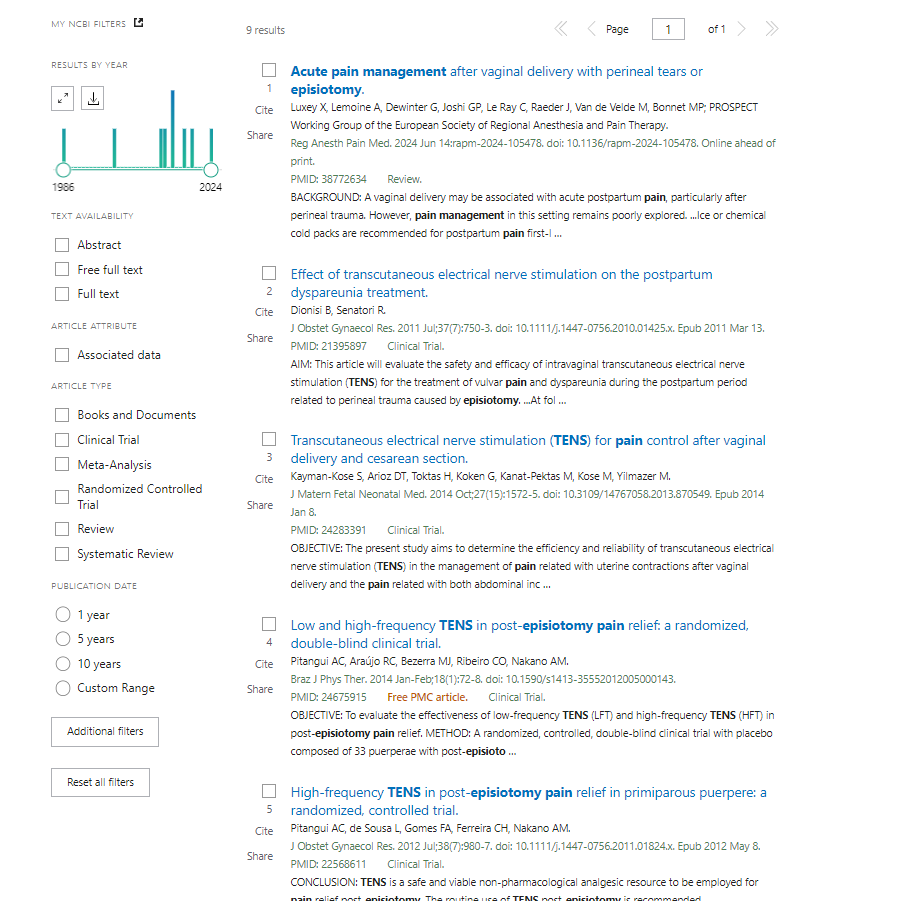


CINHAL


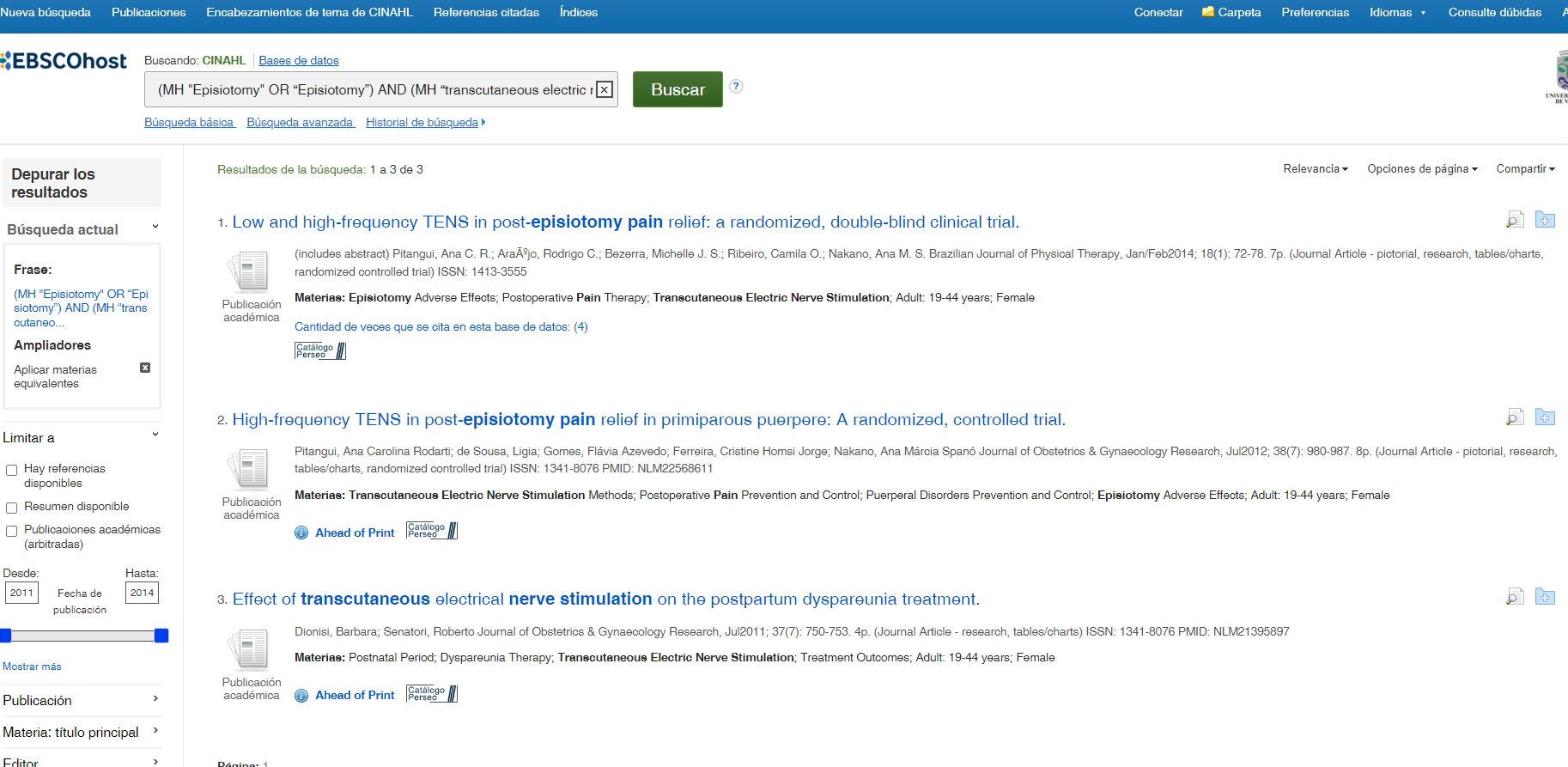


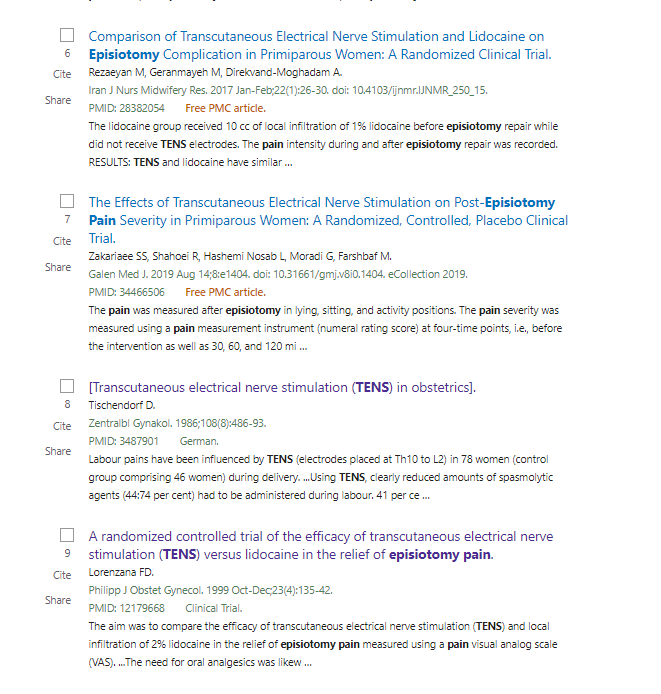


Scopus


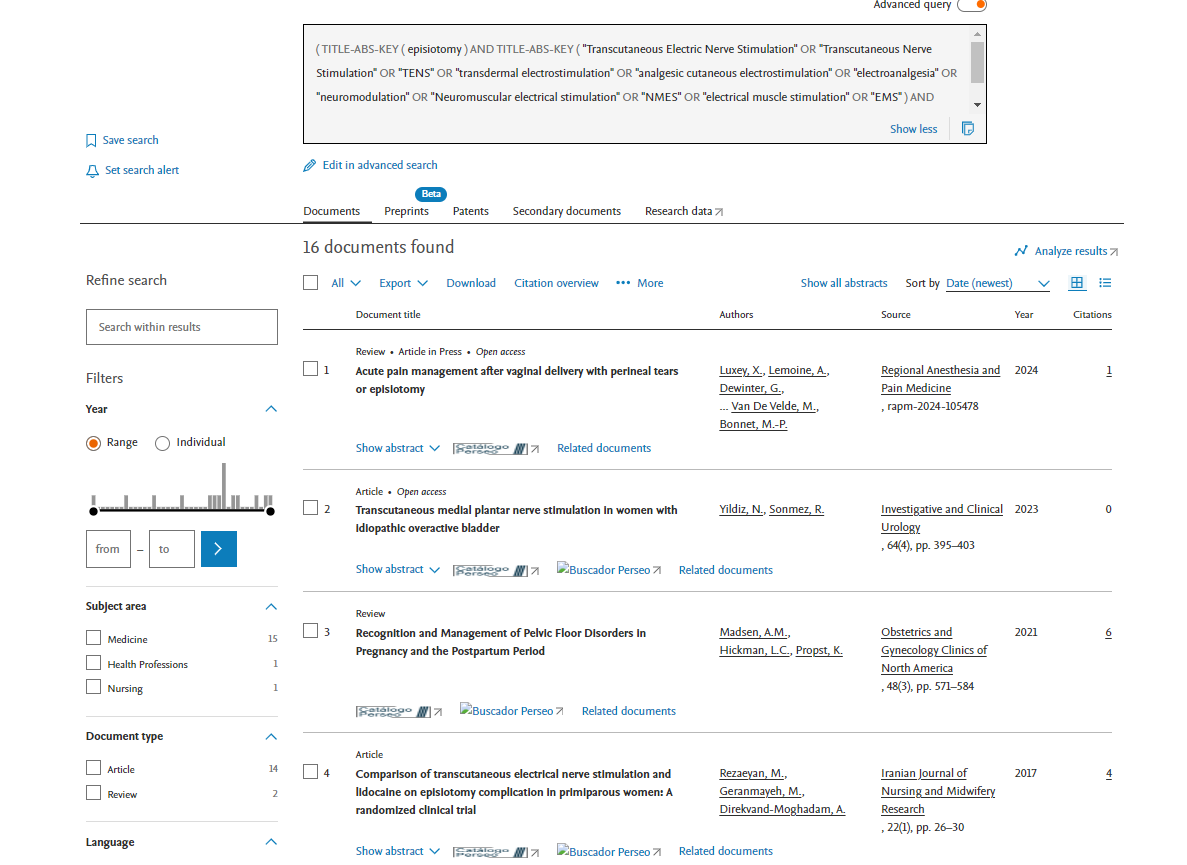


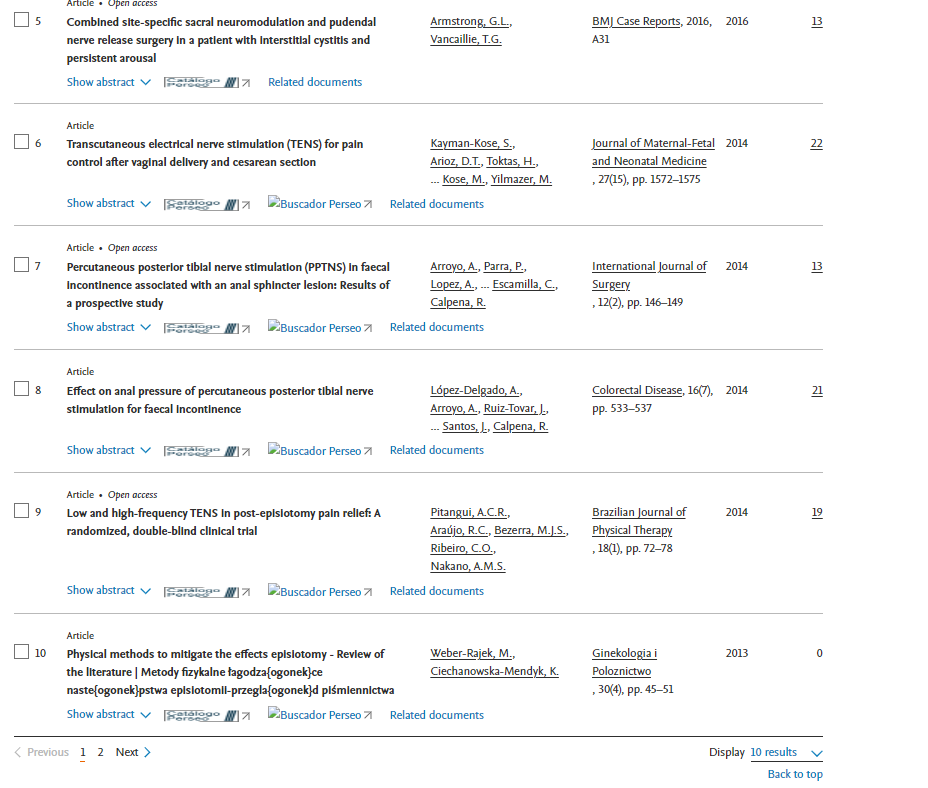


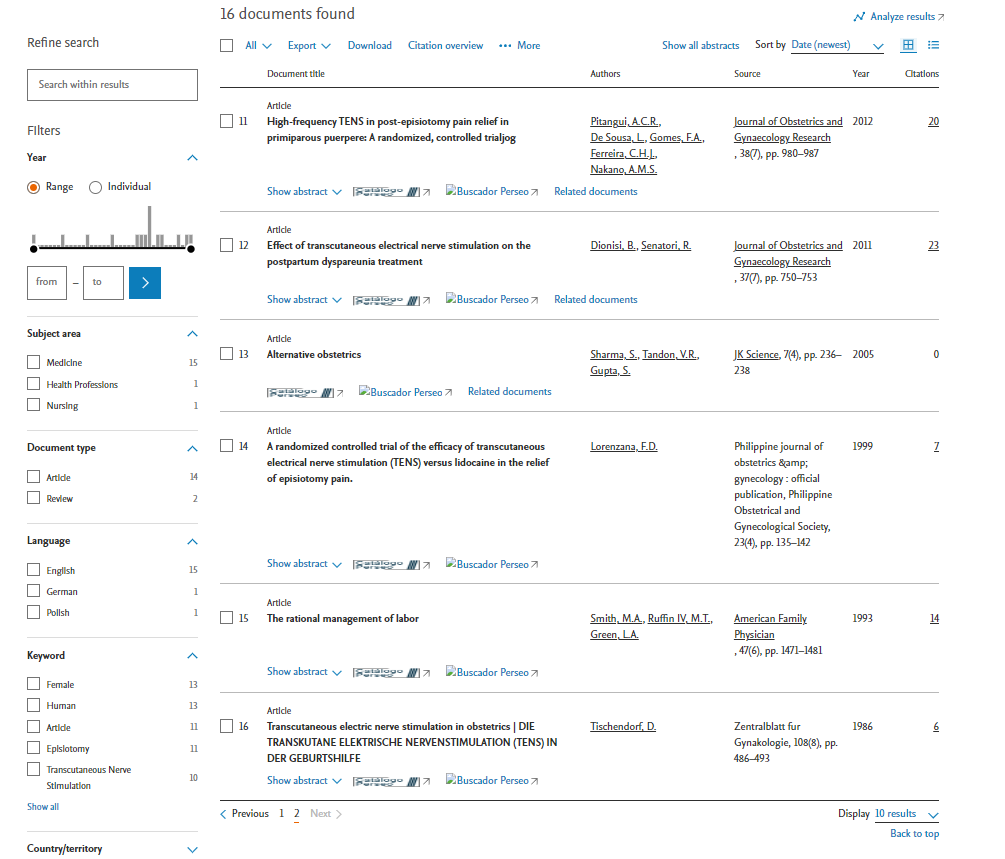


Cochrane


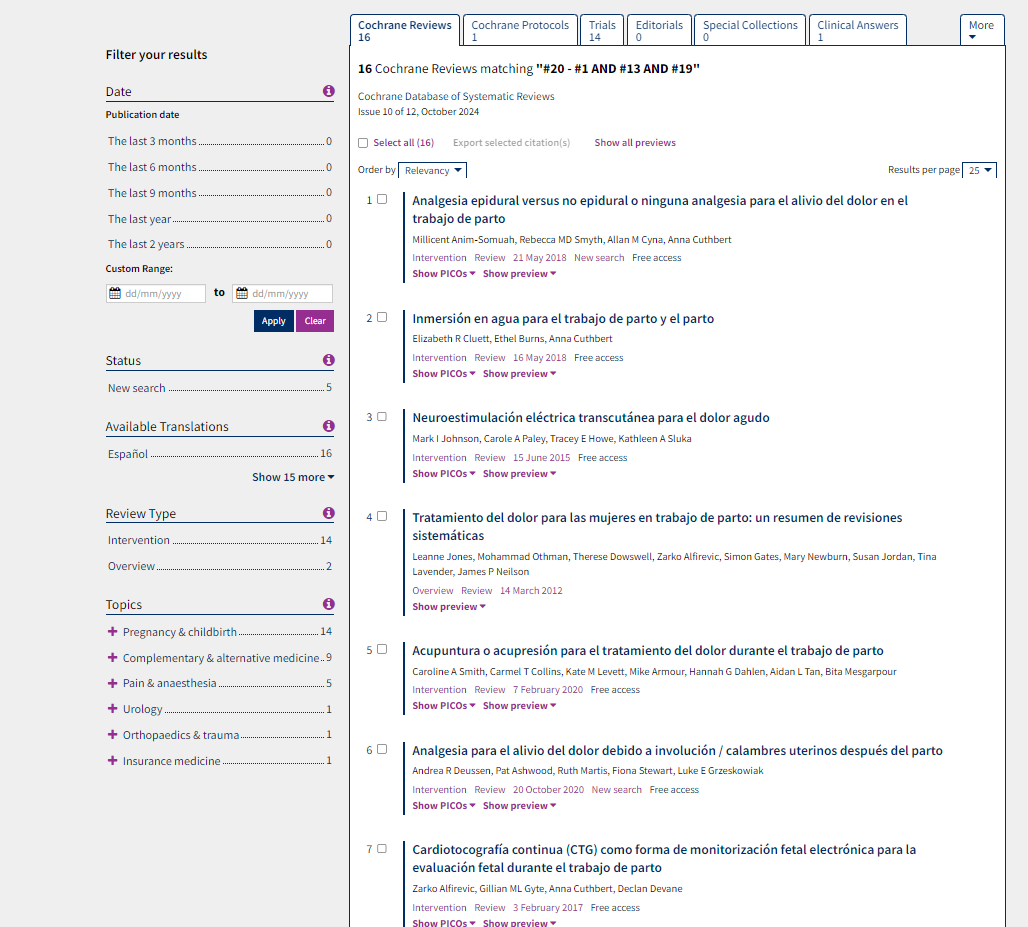


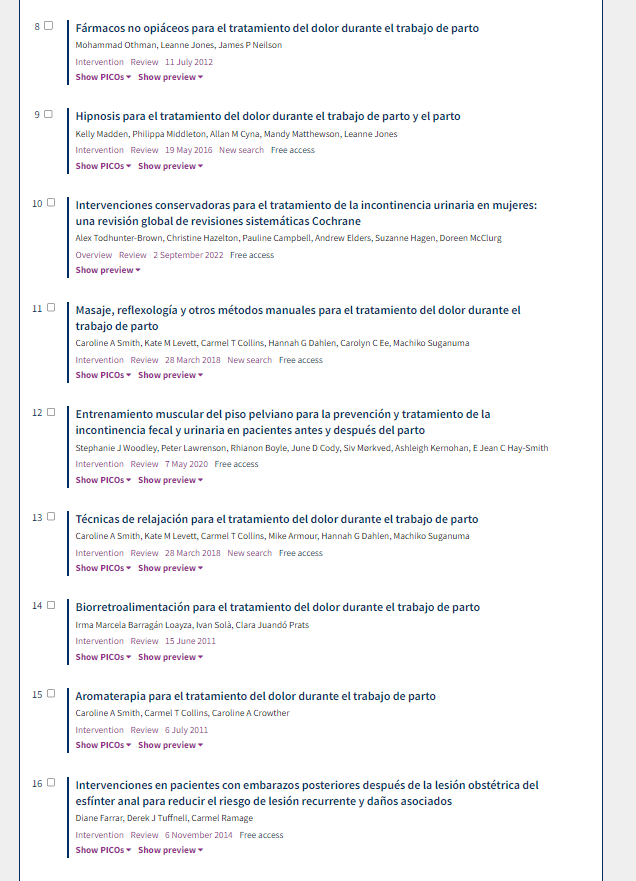


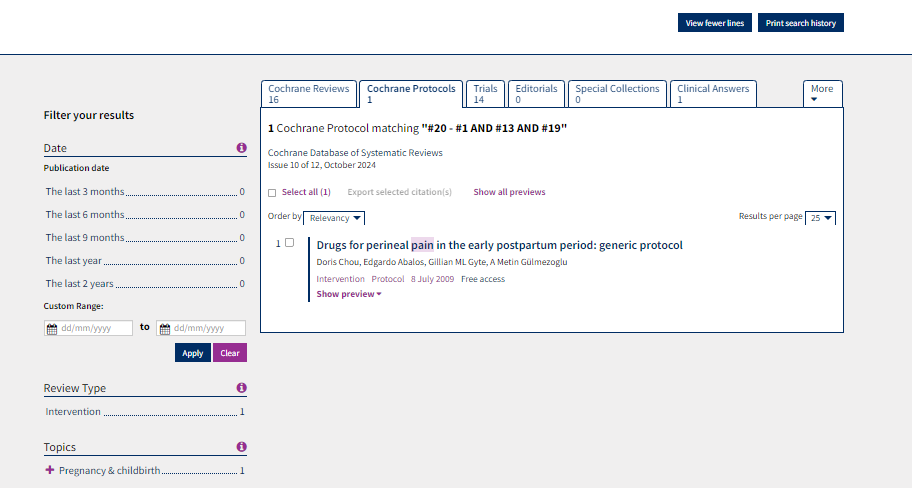


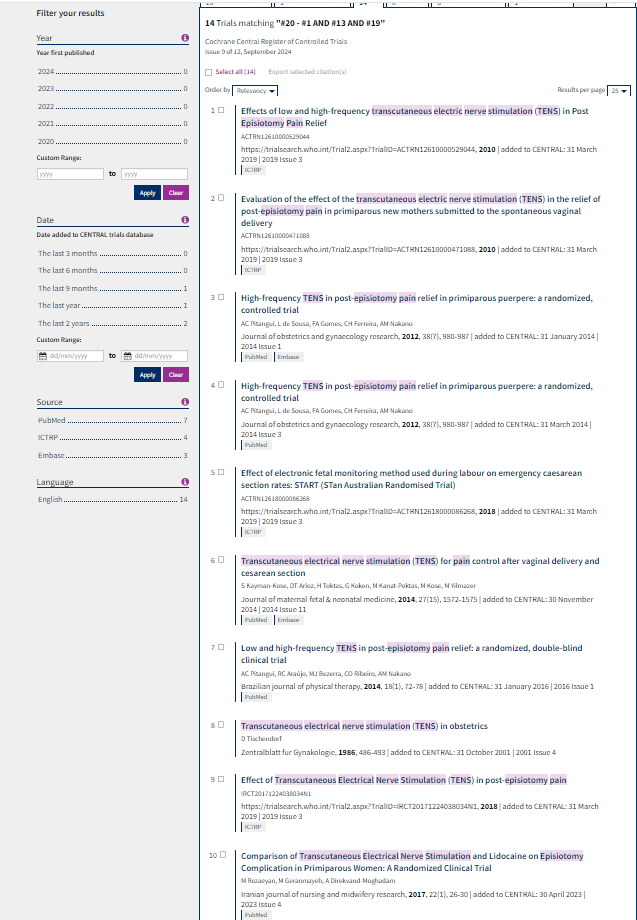


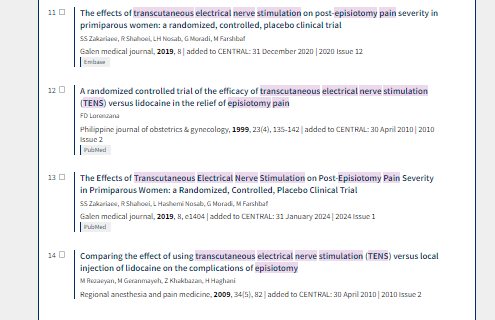


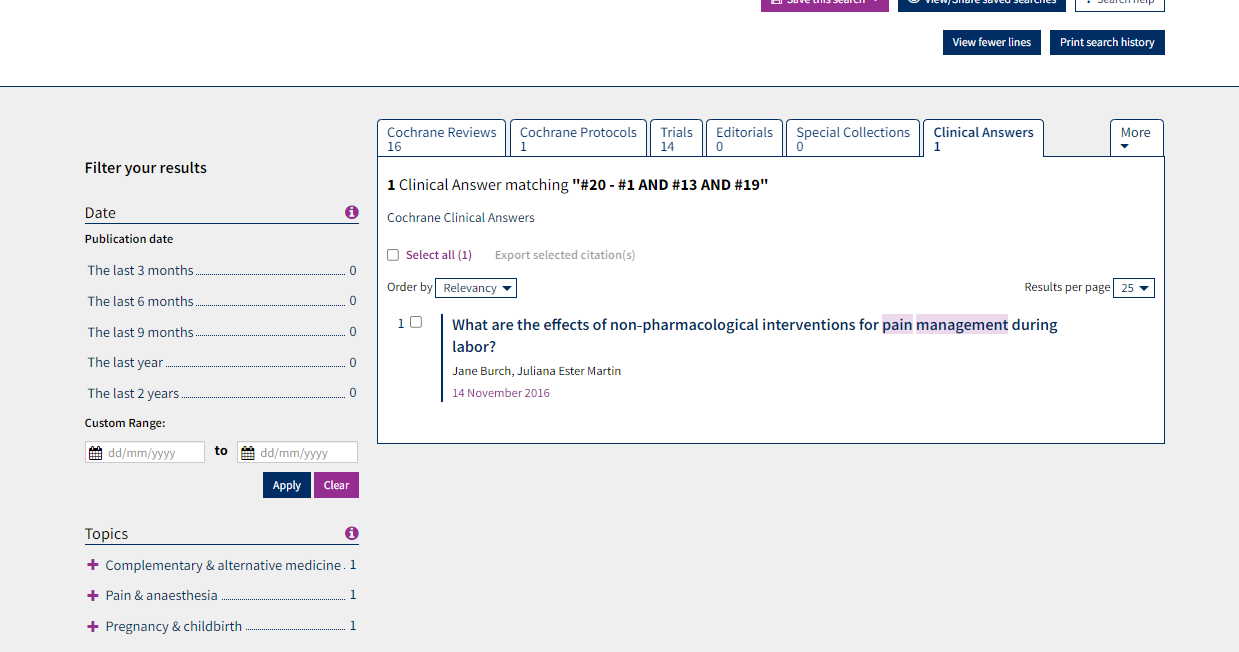


Web of Sciences


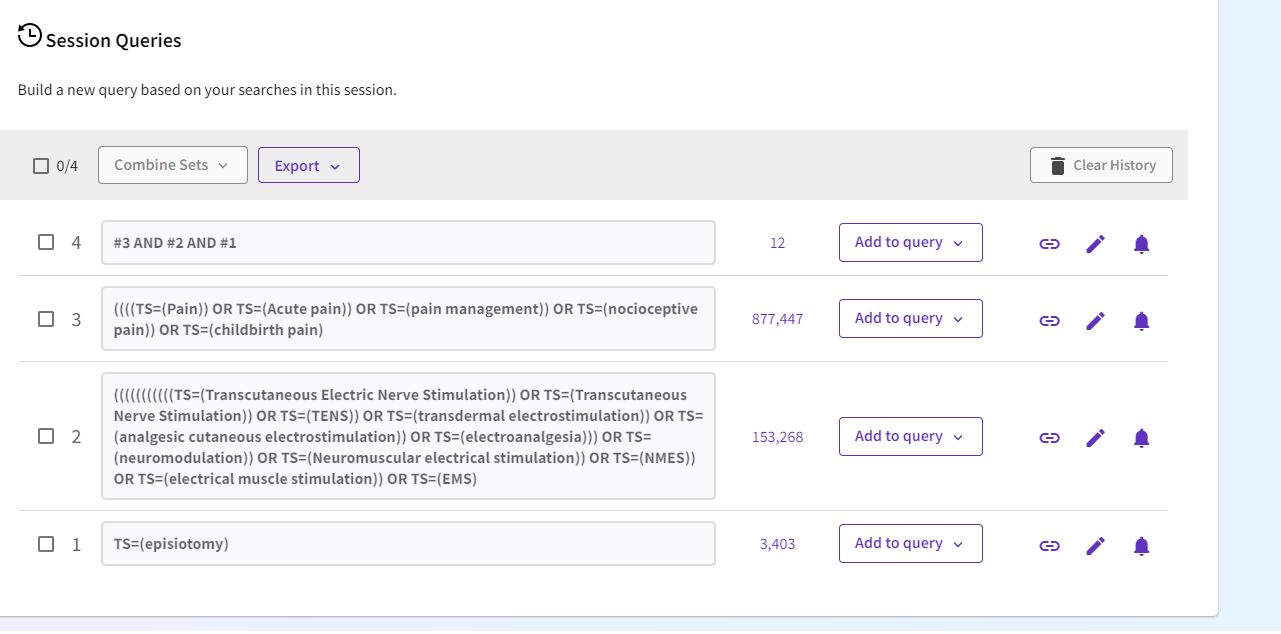


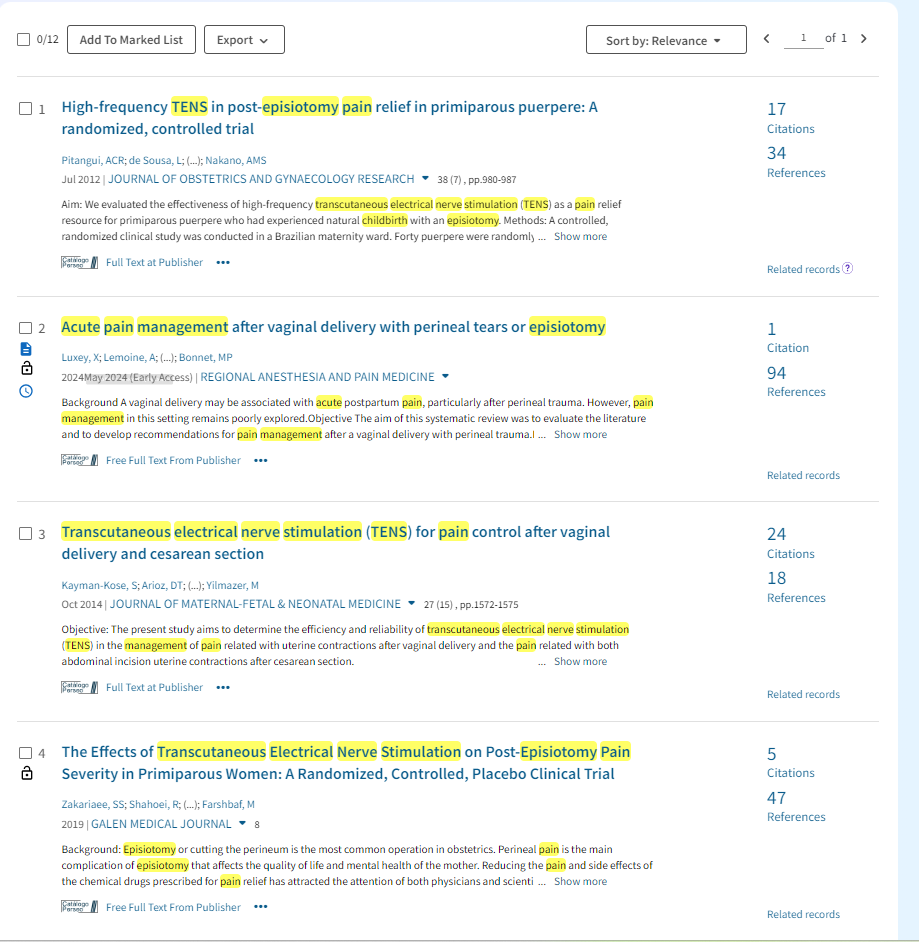


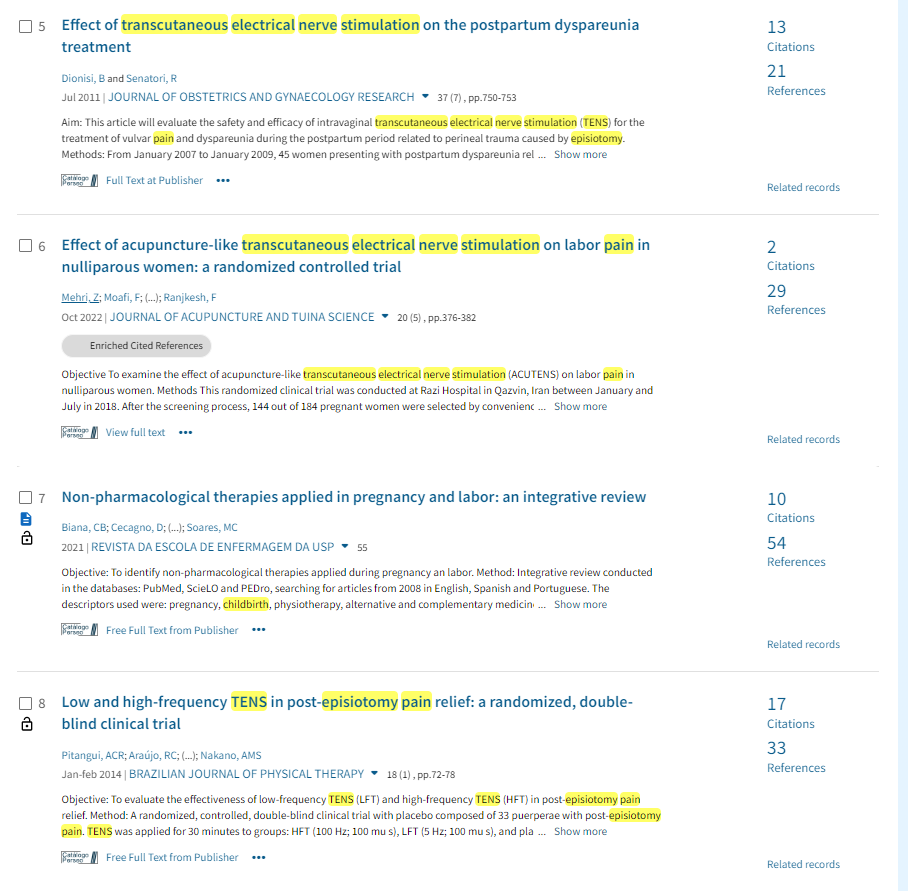


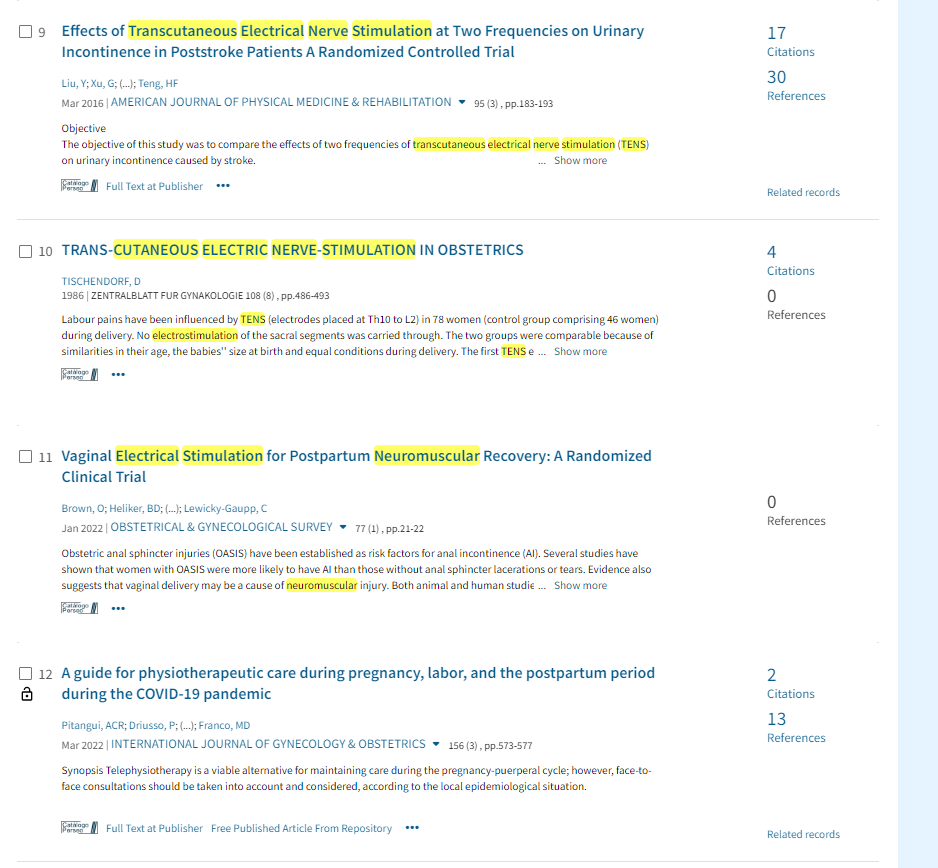


PEDro


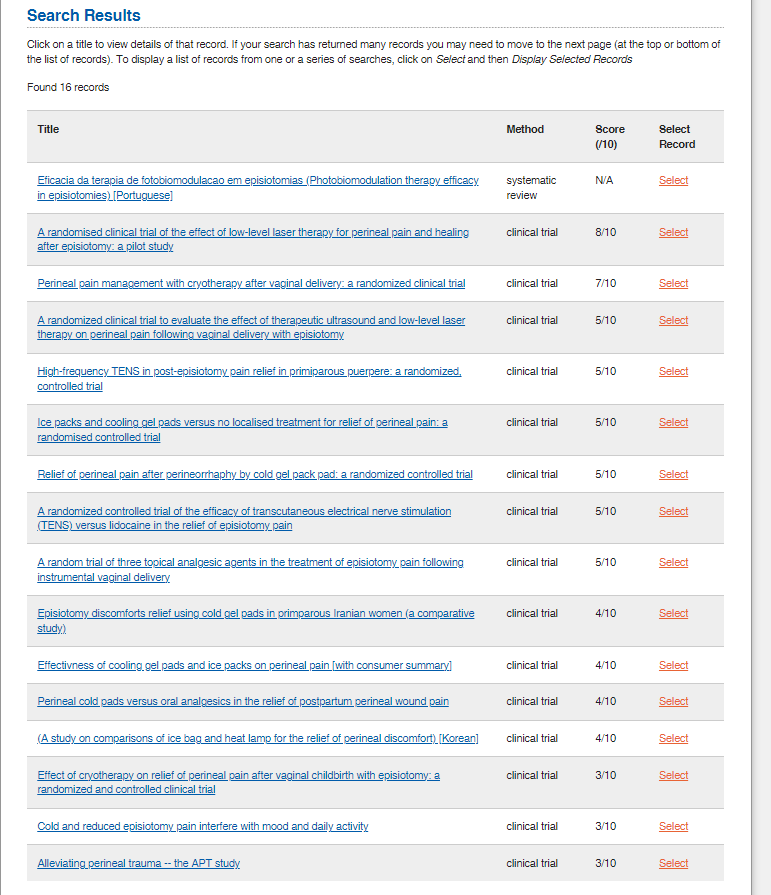


1. **DUPLICATES REMOVED FROM ZOTERO**


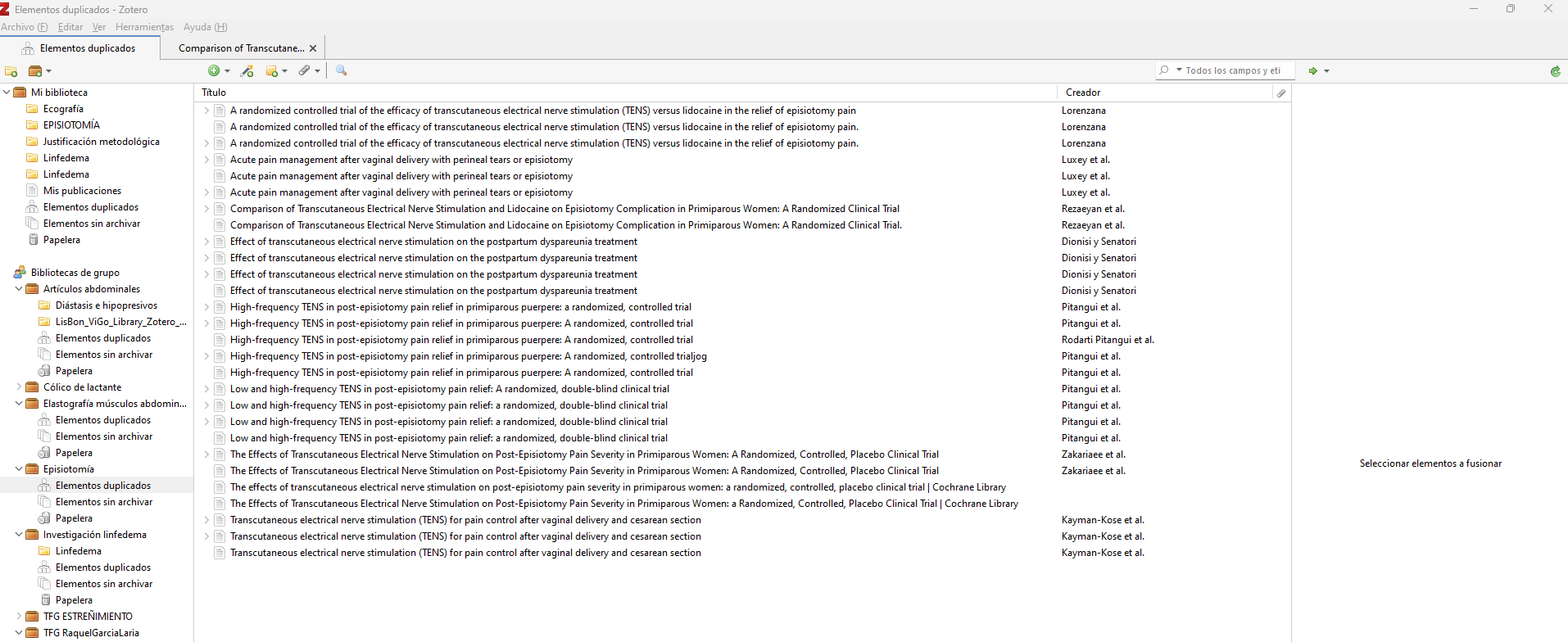

Supplement: Multimedia component 1 [file mmc1.doc]
